# High ACSL5 Transcript Levels Associate with Systemic Lupus Erythematosus and Apoptosis in Jurkat T Lymphocytes and Peripheral Blood Cells

**DOI:** 10.1371/journal.pone.0028591

**Published:** 2011-12-06

**Authors:** Antonio Catalá-Rabasa, Dorothy Ndagire, Jose Mario Sabio, Maria Fedetz, Fuencisla Matesanz, Antonio Alcina

**Affiliations:** 1 Department of Cell Biology and Immunology, Instituto de Parasitología y Biomedicina “López Neyra”- Consejo Superior de Investigaciones Científicas (IPBLN-CSIC), Granada, Spain; 2 Unidad de Enfermedades Autoinmunes Sistémicas, Servicio de Medicina Interna, Hospital Universitario Virgen de las Nieves, Granada, Spain; University of Crete, Greece

## Abstract

**Background:**

Systemic lupus erythematosus (SLE) is a prototypical autoimmune disease in which increased apoptosis and decreased apoptotic cells removal has been described as most relevant in the pathogenesis. Long-chain acyl-coenzyme A synthetases (ACSLs) have been involved in the immunological dysfunction of mouse models of lupus-like autoimmunity and apoptosis in different *in vitro* cell systems. The aim of this work was to assess among the ACSL isoforms the involvement of ACSL2, ACSL4 and ACSL5 in SLE pathogenesis.

**Findings:**

With this end, we determined the ACSL2, ACSL4 and ACSL5 transcript levels in peripheral blood mononuclear cells (PBMCs) of 45 SLE patients and 49 healthy controls by quantitative real time-PCR (q-PCR). We found that patients with SLE had higher ACSL5 transcript levels than healthy controls [median (range), healthy controls = 16.5 (12.3–18.0) vs. SLE = 26.5 (17.8–41.7), P = 3.9×10 E-5] but no differences were found for ACSL2 and ACSL4. In *in vitro* experiments, ACSL5 mRNA expression was greatly increased when inducing apoptosis in Jurkat T cells and PBMCs by Phorbol-Myristate-Acetate plus Ionomycin (PMA+Io). On the other hand, short interference RNA (siRNA)-mediated silencing of ACSL5 decreased induced apoptosis in Jurkat T cells up to the control levels as well as decreased mRNA expression of FAS, FASLG and TNF.

**Conclusions:**

These findings indicate that ACSL5 may play a role in the apoptosis that takes place in SLE. Our results point to ACSL5 as a potential novel functional marker of pathogenesis and a possible therapeutic target in SLE.

## Introduction

Systemic lupus erythematosus (SLE) is a complex genetic autoimmune disorder which predominantly affects women and leads to the production of antibodies against an individual's own healthy tissues, aberrant formation of immune complexes (IC), and inflammation of multiple organs. As a consequence skin rashes, joint pain, anaemia, cardiovascular-atherosclerosis and renal diseases are the principal clinical manifestations. While no known cure for SLE exists, current treatments that range from antimalarials to corticosteroids and immunosuppressive agents have markedly reduced short-term mortality rates. Long-term mortality rates, on the other hand, are increasingly influenced by cardiovascular complications [1].

Accelerated apoptosis of circulating lymphocytes and/or impaired clearance of apoptotic cells are already known to be a hallmark of SLE [2]. Impaired engulfment of early apoptotic cells may cause secondary necrosis and release of intracellular autoantigens, and then trigger autoimmune reactions in SLE [3], [4]. The dysfunction of apoptosis may be a direct consequence of alterations in proteins/genes such as Fas, FasL, Bcl-2 and C1q among others [5], [6].

Human long-chain acyl-CoA synthetases (ACSL, EC6.2.1.3) activate fatty acids with chain lengths from 12 to 20 carbon atoms by esterification with coenzyme A (CoA). The acyl-CoAs formed are essential for complex lipid synthesis, protein modification, beta-oxidation, regulation of various physiological processes and remodelling of membranes [7]. ACSLs differ in fatty acid types and tissue expression preference. ACSL5 has a substrate preference for C16-C18 unsaturated fatty acids and is expressed in small intestine, as well as in lungs, liver and other tissues, localizing at the outer mitochondrial membrane and microsomes [8], [9].

Acyl-coAs are lipid metabolic intermediates that have been associated to metabolism regulation systems and gene expression [10]. Besides, acyl-coAs have long been associated to apoptosis, mostly because of their effect on membrane stability, signaling pathways and secondary metabolite activity [11]–[13].

Although the contribution of lipid metabolic pathways to autoimmunity, and specifically to SLE, is poorly understood, several *in vivo* and *in vitro* evidences indicate that ACSLs may play an important role in the immune dysfunction of lupus-like mouse models [14], [15]. In addition, autoimmunity and inflammation are associated with marked changes in lipid and lipoprotein metabolism in SLE [16], [17]. ACSL5, on the contrary, has been associated with cell development and maturation, physiopathological processes, apoptosis and tumorigenesis [18]–[21].

In spite of these suggestive precedents, this study is the first to approach a possible involvement of ACSL5 in autoimmune diseases and specifically in SLE. We hypothesized an implication of ACSL5 in the pathogenesis of SLE, which is associated with the increased apoptosis seen in the disease, since this gene has already been associated to apoptosis in other tissues and diseases. For that, we investigated the ACSL5 transcripts levels in peripheral blood mononuclear cells (PBMCs) from SLE patients and healthy donors to seek for significant differences. Moreover, we focused on whether ACSL5 transcript levels were related to activation-induced cell death (AICD) in Jurkat T cells and PBMCs as models already described [22], and discuss its potential association with apoptosis found in SLE. Our results indicated that ACSL5 transcript levels were higher in SLE than in controls and that silencing ACSL5 mRNA by short interference RNA (siRNA) decreased the apoptosis induced by phorbol-myristate-acetate plus ionomycin (PMA+Io)-activation of Jurkat T cells, thus implicating ACSL5 in AICD.

## Results

### ACSLs measurements in patients and controls

The aim of this study was to investigate the mRNA expression levels of ACSL5 in PBMCs from SLE patients and control subjects by means of qPCR. The main characteristics of the SLE patients and controls relevant for this study are listed in Table 1. We have quantified mRNA levels in two conditions. Firstly, from freshly extracted and non-activated PBMCs named as ACSL5(N), potentially indicative of physiological status of each group, either patients or controls. The second type of measurement for ACSL5 was done after 24 h of *in vitro* stimulation with PMA+Io named ACSL5(A). This kind of activation may resemble to a natural polyclonal activation conditioned by the pathological status of the individuals from whom PBMCs were extracted and may be indicative of the maximum response potential of PBMCs. Finally, a parameter that we set up to equilibrate differences owning to undetermined factors between measurements (supposing to affect similarly to the untreated and activated cultures), was the stimulation index (SI), that is, the ratio between ACSL5(A) and ACSL5(N) expression levels [23].

**Table 1:** Main characteristics of study subjects with systemic lupus erythematosus.

*General Characterisitics*	*Patients (n = 45)*
Age (yrs)^1^	42±13
Female (%)^2^	88
**SLE characteristics**	
Disease duration (yrs)	13.3±8.6
Age at SLE diagnosis (yrs)	31.6±13.1
**SLE complications**	
Arthrites (%)	90
Renal involvement (%)	28
Serositis (%)	23
Haematological manifestations (%)	33
Neurological manifestations (%)	23
Positive anti-dsDNA (ever) (%)	83
Antiphospholipid antibody (ever) (%)	28
**Activity**	
SLEDAI score	4.4±3.6
SDI score	1.23±1.46
**Treatments**	
Current use of prednisone (%)^3^	68
Current use of HCQ (%)	74
Mean current prednisone dose (mg/day)	3.6±3.1
Mean current HCQ dose (mg/day)	91±59
Immunosuppressant agents (%)	33

1 Age of Control group: n = 49, mean ± SD = 36.91 ±14 (19–61). No significant difference with SLE group (P-value> 0.05).

**Figure 1:**
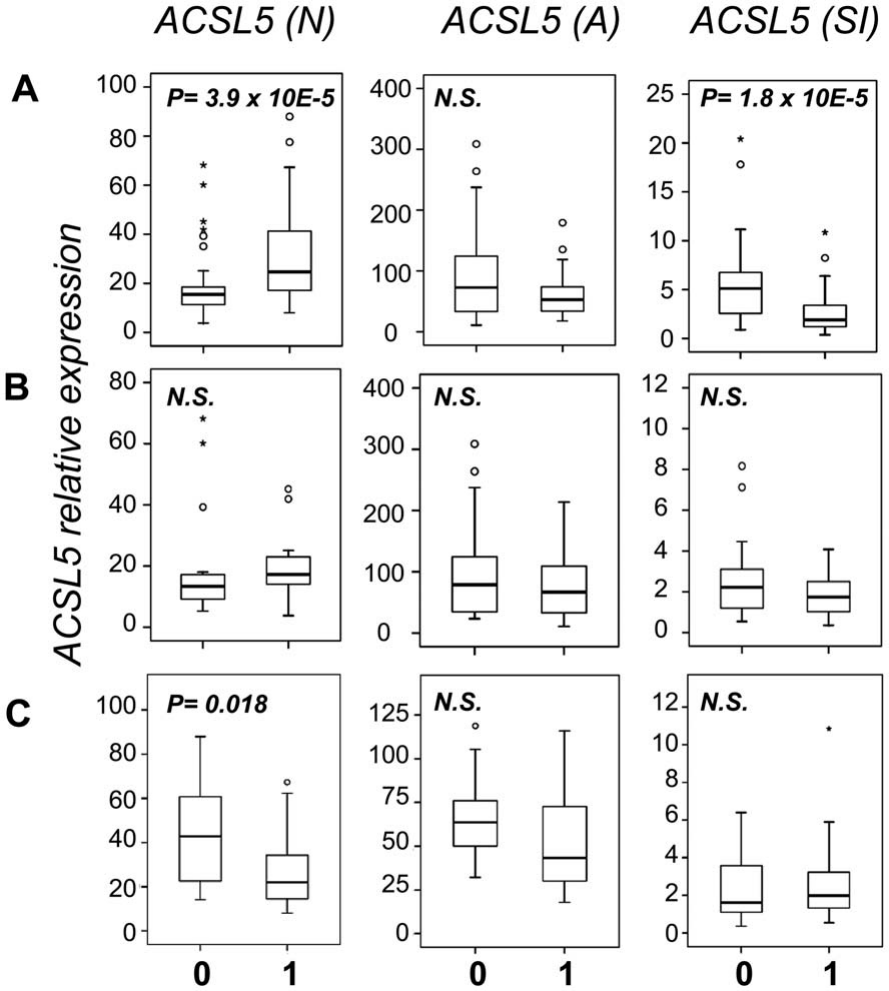
Association of ACSL5 mRNA expression levels with SLE. A) ACSL5 transcript levels in PBMCs from healthy controls (0) and SLE patients (1) for ACSL5(N) (number of samples n: 0 = 49, 1 = 45), activated ACSL5(A) (n, 0 = 34, 1 = 40) and the ratio A/N (ACSL5(SI) (n, 0 = 34, 1 = 40). B) ACSL5 transcript levels in PBMCs from males (0) (n = 24) and females (1) (n = 25), all of them from the control group. C) ACSL5 transcript levels in PBMCs from prednisone treated (1) (n = 28) and untreated (0) (n = 12) SLE patients. Results are represented in box plots given medians, quartiles, outsiders (circle points) and extremes (asterisk points). *P-values* of differences between the groups (0) and (1) are defined by Mann-Whitney Statistics. *N.S*. stands for non significant.

2 Female percentage of control group: 50%. No difference of ACSL5 levels between male and females of control group (see Figure 1B).

3 Prednisone treatment to patients affected ACSL5 transcript levels (see Figure 1C).

### ACSL5 in SLE patients vs. healthy controls

As shown in Figure 1A, comparing with healthy subjects, freshly collected PBMCs from SLE patients had higher expression levels of mRNA for ACSL5 than controls [median (range); CTL = 16.5 (12.3–18) vs. SLE = 26.5 (17.8–41.7), P = 3.9×10^−5^]. No significant difference was found for ACSL2 and ACSL4 between healthy controls and SLE patients (unpublished data). In activated PBMCs, there were no differences between expression levels from SLE patients and controls. ACSL5(SI) was significantly lower in SLE patients than in controls (median (range); CTL = 5.1 (2.5–6.8) vs. SLE = 1.9 (1.2–3.4), P = 1.8×10^−5^), suggesting an enhanced transcription of ACSL5 in SLE patients that was close to maximum state of provoked activation.

### ACSL5 difference between male and female healthy controls

While SLE group had similar age to the healthy control group, the male to female ratio was clearly different, so we took this last characteristic into account and presented experiments to determine gender influence into ACSL5 transcript levels. As shown in Figure 1B, ACSL5(N), (A) and (SI) values were similar in male and female groups of healthy controls, therefore it was deduced that gender was not associated with differences of ACSL5 transcript levels and hence we could establish that there were no differences between SLE patients and controls.

### ACSL5 differences between prednisone treated and untreated SLE patients

Another important consideration to discard confounder factors was the effect of drug treatments in the ACSL5 mRNA levels. Amongst the several drug-treatments of SLE patients indicated in Table 1, only the corticosteroid prednisone affected ACSL5 mRNA levels. Comparing transcript level differences between these two groups of SLE patients, we found a lower ACSL5(N) transcript levels in treated than untreated group as shown in Figure 1C. These results point to an even higher ACSL5 transcript levels associated with pathology if patients were not treated with prednisone which would follow our previous results referring to higher ACSL5 expression in SLE patients than in healthy controls.

### ACSL effect in SLE and as diagnostic marker

To determine the effect on SLE (odds ratio, OR), logistic regression analysis was performed showing the beta coefficient and odds ratio with 95% confidence intervals (Table 2). ACSL5 (SI) had an OR of 0.68 (95% CI = 0.544−0.854) for each increasing unit variation. Analysis of the ROC curves for ACSL5 from PBMCs showed that the AUC was highest for ACSL5 (SI) (AUC  = 0.776) as indicated in Table 2, which reflected a potential utility in diagnosis.

**Table 2:** Logistic regression analysis to determine the effect on diseases and the area under curve (AUC) of ACSL5 values in PBMCs.

*ACSL5*	*Coefficient*	*Wald*	*P-value*	*OR, 95% CI*	*AUC*
***ACSL5(N)***	0.047	7.26	0.007	1.048 (1.013–1.084)	0.732
***ACSL5(A)***	−0.011	5.316	0.021	0.989 (0.980–0.998)	0.598
***ACSL5(SI)***	0.384	11.138	0.001	0.681 (0.544–0.854)	0.776

Results are represented with the beta coefficient and the odds ratios *(OR)* with 95% confidence intervals *(CI).*

### Effect of PMA+Io activation in PBMCs

**Figure 2:**
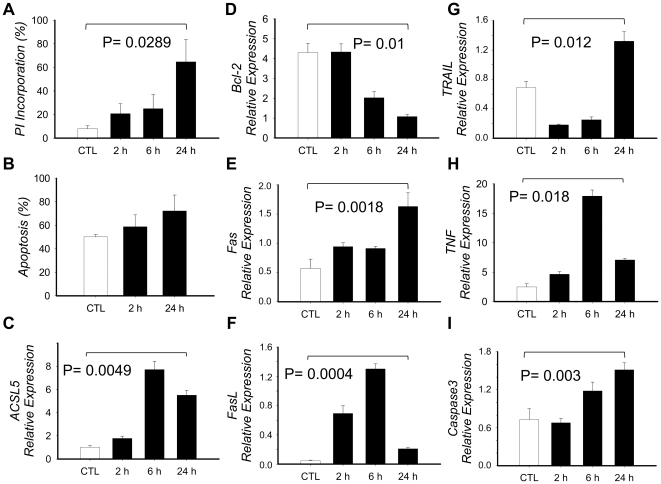
Effect of PMA+Io activation in PBMCs. PBMCs from healthy controls were obtained and cultured in the presence or absence of PMA+Io for up to 24 h. A) Cells were collected at defined times, washed with PBS, fixed with 70% Ethanol, stained with Propidium Iodide (PI) and analyzed in a FACSCaliburTM flow cytometer to determine the apoptotic hipodiploid cell fragments (defined as percentage of PI incorporation). B) Cells were washed twice with PBS and double stained with Annexin V and PI, then analyzed by FACS to determine the percentage of apoptotic cells (Annexin V positive and double positive cells). C–I) Total RNA was extracted, cDNA synthesized and qRT-PCR implemented to determine mRNA expression. Results are given by means of three independent experiments and the bars show the standard deviation. *P-value* has been calculated with the paired Student *t* test.

We obtained PBMCs from two controls and cultured them with or without PMA+Io up to 24 h followed by PI alone or Annexin V/PI double staining as described in material and methods (Figure 2 A–B). Results showed more than 50% of PI incorporation after 24 h of activation with PMA+Io compared to controls (P = 0.0289). Annexin V/PI double staining showed no significant differences compared to controls due to an abnormally elevated apoptosis in controls. We analyzed then mRNA expression of several relevant genes associated with apoptosis to partly determine the effect of PMA+Io in the apoptotic pathway (Figure 2 D–I). BCL-2 showed a 4-fold decrease, while FAS, FASLG, TRAIL (TNFSF10), TNF and CASP3 increased by 3-, 5-, 2-, 3- and 2-fold respectively, thus corroborating and induction of AICD in PBMCs by PMA+Io activation.

On the other hand, ACSL5 mRNA levels increased in a time-dependent course when activated with PMA+Io (mean±SD; 24 h = 5.51±0.41 vs. CTL = 1.02±0.13; P = 0.0049) (Figure 2C), thus correlating with the expression of pro-apoptosis genes and the induction of apoptosis in PBMCs.

### Effect of PMA+Io activation on AICD in Jurkat T cells

**Figure 3:**
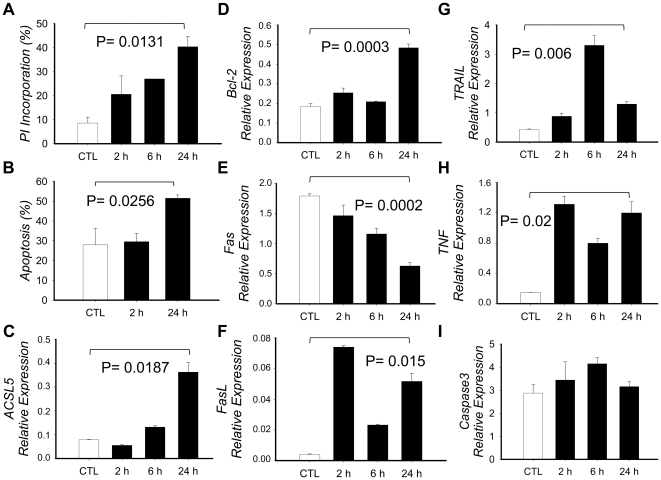
Effect of PMA+Io activation in Jurkat T cells. Jurkat T cells were cultured in the presence or absence of PMA+Io for up to 24 h. A) Cells were collected at defined times, washed with PBS, fixed with 70% Ethanol, stained with Propidium Iodide (PI) and analyzed in a FACSCaliburTM flow cytometer to determine the apoptotic hipodiploid cell fragments (defined as percentage of PI incorporation). B) Cells were washed twice with PBS and double stained with Annexin V and PI, then analyzed by FACS to determine the percentage of apoptotic cells (Annexin V positive and double positive cells). C–I) Total RNA was extracted, cDNA synthesized and qRT-PCR implemented to determine mRNA expression. Results are given by means of three independent experiments and the bars show the standard deviation. *P-value* has been calculated with the paired Student *t* test.

Jurkat T cells were activated with PMA+Io for up to 24 h, followed by PI alone or Annexin V/PI double staining (Figure 3A–B). PI positive cells in PMA+Io-activated Jurkat cells increased to 20, 27 and 40% at 2, 6 and 24 h respectively, whereas it was 8% in untreated control cells. On the other hand, Annexin V positive cells increased 2 and 23% at 2 and 24 h, respectively, compared with controls (P = 0.0256). These results agree with several studies describing PMA+Io-induced AICD in lymphocytes [22]. On the other hand, Annexin V positive cells increased, compared to controls, 2 and 23% at 2 and 24 h, respectively. We analyzed, as with PBMCs, mRNA expression of several relevant genes associated to apoptosis to partly determine the effect of PMA+Io in the apoptotic pathway (Figure 3 D–I). FASLG, TNF and TRAIL (TNFSF10) were induced 14, 8 and 3 times respectively, thus agreeing with cytometry results.

### Effect of PMA+Io activation in ACSL5 mRNA expression

Jurkat T cells activated with PMA+Io showed an increased level of ACSL5 mRNA – time dependent – by approximately 5-fold at 24 h compared to untreated control cells (mean±SD; PMA+Io = 0.36±0.04 vs. CTL = 0.079±0.002; P = 0.018) (Figure 3C), so indicating that ACSL5 is inducible by PMA+Io in Jurkat T cells. Here we concluded that the increased ACSL5 transcript levels correlated with PMA+Io-induced apoptosis in Jurkat cells.

### Effect of ACSL5 transcript levels in PMA+Io-inducing apoptosis

**Figure 4:**
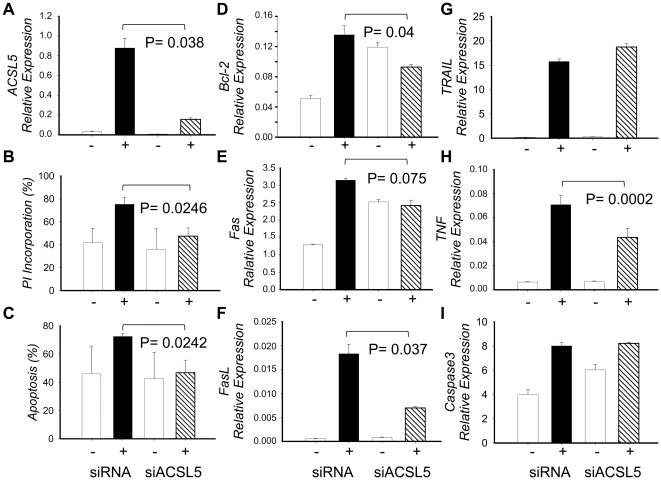
Effect of ACSL5 silencing in the apoptosis induced in Jurkat T cells by PMA+Io activation. Jurkat T cells were electropororated with either siRNA for ACSL5 (siACSL5) or unspecific control of siRNA (siRNA), and left for 18 h in culture media. Then both siACSL5 and siRNA Jurkat T cells were cultured with or without PMA+Io for 24 h. A) ACSL5 expression at 24 h was determined in each type of culture as described in material and methods. B) Percentage of apoptosis in siACSL5 or siRNA Jurkat T cells measured by cytometry as Annexin V positive and double Annexin V and PI positive cells. C–I) Total RNA was extracted, cDNA synthesized and qRT-PCR implemented to determine mRNA expression. Results are given by means of three independent experiments and the bars show the standard deviation. *P-value* has been calculated with the paired Student *t* test.

We used short interference RNA (siRNA) technology to silence ACSL5 gene expression to test whether it was implicated in AICD in Jurkat T cells. For that purpose siACSL5 and a transfection control (siRNA) were introduced by electroporation into Jurkat T cells, obtaining 80% silencing approximately after 18 h of transfection (Figure 4A). Silencing was particularly important in PMA+Io-activated cells (mean±SD; siACSL5 = 0.034±0.0005 vs. siRNA =  0.19±0.08; P =  0.038) due to a low expression level of ACSL5 in untreated cells but high expression following PMA+Io activation.

We then proceeded to test ACSL5 implication in PMA+Io-induced apoptosis in Jurkat T cells by FACS (Figure 4 B–C). Following PI alone or Annexin V/PI double staining, analysis of transfected Jurkat samples showed a 35% decrease approximately in both experiments – lowering to control levels – in siACSL5 compared to siRNA transfected Jurkat T cells when they were activated with PMA+Io (mean±SD; PI Incorporation: siACSL5 =  47±7 vs. siRNA =  75±6; P =  0.0246. Annexin V positive cells: siACSL5 =  47±9 vs. siRNA =  72±2; P =  0.0242). It is important to note that there was an elevated apoptosis in controls – and possibly in stimulated too – due to the necessity to stimulate cells with PMA+Io right after transfection because of short life of siRNAs, and so the impossibility to eliminate dead cells from the nucleofection process.

Following prior experiments we analyzed mRNA expression of several apoptotic genes, finding that FAS, FASLG and TNF decreased 1.3-, 2.6- and 1.6-fold in siACSL5-transfected cells compared to controls after stimulation with PMA+Io. These findings imply a direct role of ACSL5 in PMA+Io-induced apoptosis in Jurkat cells.

## Discussion

The aim of this study was to investigate the role of ACSL5 in SLE pathogenesis. We found that ACSL5 transcript level was significantly increased in PBMCs from SLE patients compared to controls. This association was not influenced by the different female/male ratio of the cohorts since they did not show ACSL5 differences. The stimulation index (SI), which equilibrates undetermined individual differences, showed a significantly greater stimulation increase in PBMCs from controls compared to SLE patients. Taken that SLE patients present a persistent state of activation of lymphocytes with oversecretion of pro-inflammatory cytokines [24], we could suggest that ACSL5 mRNA was overexpressed in SLE patients as a consequence of this chronic activated state of cells and thus exert a role in lymphocyte activation. Unfortunately, we could not establish a relationship between ACSL5 and cytokine expression in hematopoietic cell lines (unpublished data). In this way, a higher degree of apoptosis in T-lymphocytes in SLE patients has been found to be directly correlated with disease activity [4]. In addition, we observed that corticosteroids, used to reduce disease activity and systemic inflammation in SLE, decreased ACSL5 transcript levels, suggesting a strength link between inflammation (disease activity), apoptosis and ACSL5 levels.

There is little information about ACSL expression in PBMCs and diseases. One interesting evidence come from a microarray gene expression study of about 10,000 genes in PBMCs from 7 rheumatoid arthritis (RA) patients with rheumatoid factor (RF), 6 without RF and 7 healthy individuals. Though it showed no significant differences between RF-positive and RF-negative patients, comparisons of gene expression patterns from all RA patients and healthy controls identified a subset of discriminative genes, amongst others, a significantly higher expression in RA patients of the fatty-acid-Coenzyme A ligase (corresponding with an ACSL, although specific isoform is undetermined) together with others involved in immunoinflammatory responses, especially those related to altered phagocytic functions [25].

As shown in fas-deficient mice and humans, autoimmunity can be caused by the inability of the immune system to eliminate self-reactive lymphocytes and hence maintaining autoreactive cells that will recognise autoantibodies [5]. However, as shown in complement deficiencies, increased apoptotic material and altered clearance of apoptotic cells is found in patients with SLE [2]–[4]. This suggest that what is found in rare individuals with genetic deficiencies that develop SLE or SLE-like disease may be found in the larger population of SLE patients as a common end point pattern of unbalanced process of both apoptosis and clearance of apoptotic material. The dysfunction of apoptosis may be a direct consequence of alterations in proteins/genes such as Fas, FasL, Bcl-2 and C1q [6]. On the other hand, ACSL5 has previously been associated to both apoptosis and surviving processes of tumors from different tissues [20], [26], thereby implying a role of ACSL5 in these pathways. In addition, it has been described in SLE patients an augmented spontaneous apoptosis of lymphocytes [5], which, in the same line with our findings in patients, points to ACSL5 as a possible key molecule regulating apoptosis in SLE, and hence exerting a role in SLE pathogenesis.

Our results from experiments *in vitro* confirmed a pro-apoptotic role of ACSL5 in AICD occurring in lymphocyte cells, as this had been previously demonstrated in hepatocytic cells, increasing TNF- and TRAIL-mediated apoptosis [20]. Unlike this hypothesis, other works have shown enhanced ACSL5 expression associated to the development of colorectal cancer [21] and involved in surviving of glioma cells under acidosis conditions [26], pointing to an anti-apoptotic role. Taken together, ACSL5 may be able to play both pro- and anti-apoptotic roles depending on tissue specificity, physiopathology conditions and other factors, thus contributing to different clinical manifestations appearing in complex diseases as SLE.

Apoptosis is a genetically controlled process initiated by two principal pathways. The extrinsic pathway is activated by the ligation of death receptors, and the intrinsic pathway emerges from the mitochondria [5]. Lipids have long been associated to apoptosis through lipid-peroxidation and mitochondrial permeability transition [12], and, even though poorly understood, there have been described marked changes in lipoproteins and triglycerides in SLE patients [17]. Oxidized fatty acids found augmented in sera from SLE patients include hydroxyls and aldehydes that are linked to modification of lipids, proteins and DNA; moreover, immunization with oxidatively modified autoantigens accelerate disease progress in MLR/lpr mice [27], [28]. ACSLs are essential for lipid metabolism (synthesis and degradation) and acyl-modification of cellular components, mediated by its enzymatic activation of fatty acids [7]. This, consequently, points to these enzymes as possible regulators of these processes.

ACSL5 localizes in the mitochondria and microsomes [8], [9], from where it may execute its role in apoptosis, probably by supplying fatty acids into the mitochondria, where they can participate in oxidative reactions, increase ceramide synthesis and affect the mitochondrial membrane potential [11], [12], [27]–[29]. Lipid-peroxidation is augmented in SLE and correlates with disease activity [30], [31], and has also been associated to exposition of oxidized phosphatidylserine in the plasma membrane, associated with immunity and apoptosis due to recognition by macrophages [32]. Overexpression of ACSL5 in SLE patients may be an important key in predisposition and progression of autoimmunity; pointing to the involvement of this enzyme in supplying the material necessary for oxidative modification of cellular and extracellular components in early stages of SLE and SLE-like diseases, being ACSL5 directly associated with apoptosis and thus leading to an augmented apoptosis and recognition of these modified antigens by macrophages, which in the end could activate lymphocytes and promote autoantibody production.

In conclusion, our findings point to ACSL5 as a key regulator of AICD in lymphocytes, playing a pro-apoptotic role. We have found that silencing ACSL5 decreases FAS, FASLG and TNF expression. This supports the hypothesis that this enzyme is implicated in SLE pathology not only by directly mediating the spontaneous apoptosis occurring in SLE but also indirectly involved in presenting self-antigens to immune cells and hence, promoting the pro-inflammatory state for the predisposition and progression of autoimmune diseases such as SLE. And probably by inducing TNF expression among other pro-inflammatory genes. Future perspectives should take into account the knowledge of molecular contribution of ACSL5 in the apoptotic pathway. We therefore propose ACSL5 as a diagnostic marker and potential therapeutic target for SLE.

## Materials and Methods

### Participants

Forty five Caucasian patients (39 female, 6 male), fulfilling the American College of Rheumatology criteria for SLE [33], who attended the out-patient clinic of the University Hospital Virgen de las Nieves of Granada, Spain, were included (Table 1). Exclusion criteria were less than 1 year of follow-up in our unit. Current clinical assessment were made during a routinely visit and other demographic and clinical data were obtained from the medical records in a computer database. Disease activity and accumulated organ damage were measured with the SLE Disease Activity Index (SLEDAI) and the Systemic Lupus International Collaborating Clinics/ACR Damage Index (SDI) respectively as indicated in Table 1. Controls were healthy individuals attended to the Blood Bank of Granada. Mean age of this group was similar to the SLE group (P>.05) but the % of females (49%) was rather different to the SLE group (88%). So, we tested the effect on the ACSL5 transcript levels of males and females as indicated in Table 1.

### Ethics Statment

All participants provided a written informed consent to participate in this study, which was approved by the Institutional Review Board of Hospital Virgen de las Nieves of Granada, Spain.

### Cell cultures

Jurkat T cells (Clone E6-1, LGC/ATCC^R^) and peripheral blood mononuclear cells (PBMCs) from anonymous donors in the Blood Bank of Granada, Spain, were cultured in RPMI 1640 complete medium (PAA Laboratories, GmbH), supplemented with 10% (v/v) heat-inactivated fetal bovine serum, 2 mM (w/v) glutamine, and 100 U/mL penicillin and 100 ug/mL streptomycin (all from Gibco, Invitrogen. Carlsbad, CA), at 37°C / 5% CO_2_ atmosphere. Cells were maintained in an exponential growth phase for all experiments. To study effect of Activating-Induced Cell Death (AICD), 50 ng/mL Phorbol 12-Myristate 13-Acetate (PMA) and 10 ng/mL Ionomycin (Io) (Sigma-Aldrich Inc.) were added to the cultured cells for different-time experiments. Viable-cell counting was carried out by Trypan Blue solution (Sigma-Aldrich Inc.) staining.

### ACSL5 transcript measurement

PBMCs were obtained from whole blood samples by Ficoll-Hystopaque (Sigma-Aldrich Inc.) density centrifugation. Total RNAs were isolated by using the RNeasy mini-kit (Qiagen, Valencia, CA) and the mRNA purified by using the GenElute mRNA Miniprep kit (Sigma-Aldrich Inc.) and stored at −80°C until used.

cDNA was synthesized using a total of 200–500 ng mRNA of each sample, reverse transcribed using the Superscript III reverse transcription reagents (Invitrogen S.A., Invitrogene Ltd., UK) and then subjected to RT-PCR. As a reference gene for normalization to calculate the ACSL5 relative expression, the ubiquitin-conjugating enzyme UBcH5B was used [34]. Amplifications of cDNAs were done in triplicates using the sybr green^R^ Mastermix for real time-PCR (Biorad Laboratories, Inc.) and 50 nmol of the following primer sets: *ACSL2* Forward 5′-TTCGAAGAAGCCCTGAAAGA-3′ and Reverse 5′-AGAAATCAGCCACCACGTTC-3′ (renamed as ACSL6, ENST00000354273); ACSL4 Forward 5′- TCCAAGTTTGGGAAGAAGGA-3′ and Reverse 5′-GGCAATGGTGTTCTTTGGTT-3′ (Ensemble ID, ENST00000354273), *ACSL5* Forward: 5′ – AAGGCATTGGTGCTGATAGG – 3′ and Reverse: 5′ – TCAGGTCTTCTGGGCTAGGA – 3′ (Ensemble transcript ID ENST00000357430); and *UbcH5B as reference gene*: Forward 5′ – CAATTCCGAAGAGAATCCACAAGGAATTG – 3′ and Reverse: 5′ – GTGTTCCAACAGGACCTGCTGAACAC – 3′ (Ensemble transcript ID, ENST00000398733).

PCR conditions were as follows: 1 cycle of 95°C for 3 min, followed by 40 cycles of 95°C for 20 s, 62°C for 20s, and 72°C for 20 s. All assays were validated for linearity of amplification efficiency. PCR efficiencies were calculated using a relative standard curve derived from mixed cDNA of different samples (a twofold dilution series with four measuring points). To ensure the absence of amplification artefacts and primer dimmer formation, end point PCR products were initially assessed on ethidium bromide stained agarose gels that gave a single band of the expected size for each assay. Negative controls containing no template cDNA were run in each condition and gave no results. The reactions were quantified when the PCR product of interest was first detected (cycle threshold, CT). Calculations for relative mRNA transcript levels were performed using the comparative CT method_2_ΔΔCT_ between cycle thresholds of different reactions using Image Quant (Bio-Rad Laboratories Inc.).

### ACSL5 from non-activated (N) and activated (A) PBMCs: stimulation index (SI)

In a first step, we quantified *ACSL5* mRNA levels from freshly extracted PBMCs. The second type of measurement was done after 24 h of *in vitro* stimulation by phorbol-myristate-acetate (PMA) and ionomycin (Io). Finally, a parameter that we set up to equilibrate differences due to undetermined factors between measurements, was the stimulation index (SI), that is, the ratio between activated and non-activated expression levels [23].

### Apoptosis related mRNA expression

We quantified some relevant genes in the apoptotic pathway: BCL-2 (ENSG00000171791), FAS (ENSG00000026103), FASLG (ENSG00000117560), TRAIL (TNFSF10; ENSG00000121858), TNF (ENSG00000232810) and CASP3 (ENSG00000164305). Primers' sequences are indicated in Table S1. cDNA was synthesized using a total of 200–500 ng mRNA of each sample and amplifications of cDNAs were done in triplicates. PCR conditions were as follows: 1 cycle of 95°C for 3 min, followed by 45 cycles of 95°C for 20 s, 63°C for 20 s, and 72°C for 20 s.

### Short interference RNA (siRNA)

To silence *ACSL5* mRNA expression we electroporated Jurkat cells with specific siRNA [35]. 300 nM of siACSL5 or the transfection control (siRNA) (Dharmacon, Thermo Fisher Scientific. Lafayette, CO) were introduced into Jurkat T cells with Cell Line Nucleofector^R^ Kit V Solution Box (Amaxa, Lonza Cologne GmbH), following supplier instructions, and using the Nucleofector^R^ II (Amaxa, Lonza Cologne GmbH) electroporator. After transfection Jurkat T cells were cultured in complete medium for 18 h prior to further experiment.

### Flow cytometry

We used Propidium Iodide (PI) (Sigma-Aldrich Inc.) staining to detect hipodiploid cell fragments produced in PMA+Io-induced apoptosis [36]. Cultured PBMCs or Jurkat T cells (activated and non activated by PMA+Io for 24 h) were collected at 2, 6 and 24 h, washed twice with PBS-glucose at 2 mg/mL (VWR International, LLC. Amresco Inc., OH) and fixed with cold 70% ethanol for 15 minutes at 4°C. Then we washed cells with PBS-glucose and stained the activated and non activated cells with final concentration of 100 ug/mL Propidium Iodide (PI) prior to analysis with BD FACSCalibur™ flow cytometer (Becton, Dickinson and Company).

Annexin V-FITC kit for apoptosis (Sigma-Aldrich Inc.) was used for detection of phosphatidylserine in the outer plasma membrane as a specific apoptotic marker [37], following manufacturer's instructions. Cells were cultured for 24 h in presence or absence of PMA and ionomycin and collected at 2 and 24 h. Analyses of samples were carried out with the BD FACSCalibur™ flow cytometer.

### Statistics

Statistical analysis was performed using SPSS 15.0 for the Windows software package (SPSS, Chicago, USA). We used the nonparametric Mann-Whitney rank-sum test to test the significance of the difference in the transcript levels of ACSL5(N), ACSL5 (A) and ACSL5 (SI) between the healthy controls and SLE patients; females and males from the control group; and prednisone treated SLE patient group versus the untreated SLE group. Relative ACSL5 mRNA levels are presented as medians (range). To determine the effect on risk, we used simple logistic regression analysis and results are expressed as logistic coefficient (B), and odds ratios with the 95% confidence intervals. Receiver-operating characteristic (ROC) curves (not shown) and the area under the ROC curve (AUC) were used to assess the feasibility of using peripheral blood mononuclear cell ACSL mRNA concentration as diagnostic tools for detecting SLE. Paired Student T test was performed for comparisons between control and PMA+Io-activated and/or transfected Jurkat T cells. Errors bars are presented as mean (standard deviation) from three independent experiments. All analyses used a 2-sided level of significance of 5% (P<0.05).

## Supporting Information

Table S1 Primers used for quantification of different apoptosis associated genes.
(DOC)
