# Supplementary material for: High ACSL5 Transcript Levels Associate with Systemic Lupus Erythematosus and Apoptosis in Jurkat T Lymphocytes and Peripheral Blood Cells
Source: PLoS One. 2011 Dec 6;6(12):e28591. doi: 10.1371/journal.pone.0028591 (PMC3232234; doi:10.1371/journal.pone.0028591)
Supplement: Table S1 — Primers used for quantification of different apoptosis associated genes. (DOC) [file pone.0028591.s001.doc]

**Table S1.** Primers used for quantification of different apoptosis associated genes.

| ***Gene*** | ***Primer*** | ***Sequence (5'-3')*** |
| --- | --- | --- |
| ***BCL2*** | Forward | GTG AAC TGG GGG AGG ATT GT |
|  | Reverse | CCA GCC TCC GTT ATC CTG |
| ***FAS*** | Forward | CAA GGG ATT GGA ATT GAG GA |
|  | Reverse | TGG AAG AAA AAT GGG CTT TG |
| ***FASL*** | Forward | TGG GGA TGT TTC AGC TCT TC |
|  | Reverse | CAG AGG CAT GGA CCT TGA GT |
| ***TRAIL*** | Forward | TTC ACA GTG CTC CTG CAG TC |
|  | Reverse | CAG CAG GGG CTG TTC ATA CT |
| ***TNF*** | Forward | TGC TTG TTC CTC AGC CTC TT |
|  | Reverse | CAG CTT GAG GGT TTG CTA CA |
| ***CASP3*** | Forward | ATG GAA GCG AAT CAA TGG AC |
|  | Reverse | GCT GCA TCG ACA TCT GTA CC |
